# Correction to “Advances in the noninvasive diagnosis of melanoma—40 years beyond the ABCDs”

**DOI:** 10.3322/caac.70074

**Published:** 2026-02-19

Burshtein J, Witkowski A, Zakria D, et al. Advances in the noninvasive diagnosis of melanoma—40 years beyond the ABCDs. CA Cancer J Clin. 2026;e70065. doi:10.3322/caac.70065

Table 1 and Table 2 were incorrectly switched. Table 1, “First‐ and second‐level non‐invasive detection devices for triage of pigmented skin lesions”, should be:

Table 2, “Comparison of Dermoscopy Algorithms”, should be:

Additionally, the affiliation of Joanna Ludzik was reported incorrectly. The correct affiliation should read: Department of Bioinformatics and Telemedicine, Jagiellonian University Medical College, Krakow, Poland.

We apologize for these errors.

